# Treatment of pediatric antimuscarinic delirium with oral rivastigmine

**DOI:** 10.1093/omcr/omad096

**Published:** 2023-09-25

**Authors:** Brandtly Yakey, Varun Vohra, Amarilis Martin, Andrew M King

**Affiliations:** Department of Emergency Medicine, Detroit Medical Center, Detroit, MI, USA; Michigan Poison & Drug Information Center, Department of Emergency Medicine, Wayne State University School of Medicine, Detroit, MI, USA; Department of Pediatrics, Children’s Hospital of Michigan, Detroit, MI, USA; Department of Emergency Medicine, Detroit Medical Center, Detroit, MI, USA

## Abstract

Antimuscarinic drug toxicity is a common pediatric emergency, which produces central and peripheral symptoms. Treatment of agitation and hyperactive antimuscarinic delirium, with first-line agents like cholinesterase inhibitors or benzodiazepines, is imperative to prevent severe toxicity. Intravenous physostigmine salicylate is a cholinesterase inhibitor that is commonly used to treat central antimuscarinic delirium. Its chemical structure facilitates crossing of the blood–brain barrier. Overlapping nationwide physostigmine and benzodiazepine shortages have prompted consideration of therapeutic alternatives. Rivastigmine is a long-acting cholinesterase inhibitor with a similar chemical structure to physostigmine. It represents a potential therapeutic option for antimuscarinic delirium. Rivastigmine offers potential benefits over physostigmine including a longer duration of action, slower rate of central nervous system penetration, more favorable side effect profile, and availability in multiple formulations. A paucity of literature exists describing the use of rivastigmine for central antimuscarinic delirium. We describe the effective use of oral rivastigmine in a child with central antimuscarinic delirium.

## INTRODUCTION

Pediatric antihistamine overdoses resulting in antimuscarinic toxicity are commonly encountered by clinicians and often result in admission and significant resource utilization. The ‘anticholinergic’ or antimuscarinic toxidrome, caused by blockade of muscarinic acetylcholine receptors, leads to central and peripheral effects. Central symptoms include agitation, delirium, incoherent speech, and hallucinations [1]. Agitation and delirium are challenging to control and may result in severe sequelae including rhabdomyolysis, acidosis, aspiration, and psychological distress.

Intravenous (IV) physostigmine is used to treat central antimuscarinic toxicity. Its tertiary amine structure enables crossing of the blood–brain barrier and reversible inhibition of cholinesterase, ultimately overcoming muscarinic receptor blockade. Physostigmine demonstrated superiority in reversing antimuscarinic delirium compared to benzodiazepines [1]. The nationwide shortage of physostigmine, however, has prompted a search for effective alternative antidotes.

Rivastigmine is a long-acting cholinesterase inhibitor and is structurally similar to physostigmine. Its mechanism, structure that enables penetration of the blood–brain barrier, and pharmacokinetics make rivastigmine a potential alternative treating central antimuscarinic agitation and delirium [2]. However, few reports highlight its use to treat antimuscarinic delirium, with all relevant published cases occurring in adults.

We present a pediatric case of acute central antimuscarinic delirium with improvement following oral rivastigmine administration.

## CASE REPORT

A 7-year-old male (37 kg), with gastroesophageal reflux disease, seasonal allergies, and a reported episode of seizure-like activity without confirmation the year prior, presented to the emergency department (ED) for a suspected food-related allergic reaction. Two hours prior to presentation, his mother administered a ‘standard dose’ of an over-the-counter (OTC) liquid cough and cold medication consisting of dextromethorphan and doxylamine for a sore throat. The patient’s history was significant for self-administering medicine without adult supervision requiring hospitalization. Prior to the current presentation, he was observed entering the bedroom where the dextromethorphan/doxylamine medication was stored but it was unknown if he ingested additional doses. Upon arrival, his vital signs were: blood pressure 116/91 mm Hg, heart rate (HR) 106 beats/min, temperature 37°C, respirations 24 breaths/min, and pulse oximetry 97% on room air.

Shortly after arrival, he developed two generalized tonic–clonic seizures and received IV lorazepam 2 mg and levetiracetam 20 mg/kg with cessation of seizure activity. Post-seizure, he was tachycardic with a HR in the 130s and restless, somnolent, and not following commands. He also complained of difficulty breathing, so IV diphenhydramine 37 mg, dexamethasone 12 mg, and intramuscular (IM) epinephrine 0.3 mg were administered for potential anaphylaxis. His physical examination was negative for oropharyngeal edema, urticaria, and wheezing. Ten minutes later he developed worsening combativeness, tachycardia, incoherent speech, and hallucinations. His agitation worsened, prompting administration of IM midazolam 3 mg. His electroencephalogram and computed tomography of the brain were unremarkable.

He was admitted to the pediatric intensive care unit. He required additional IV lorazepam 2 mg and IM midazolam 1.5 mg for continued agitation 13 hours post-ED presentation. His mother reported waxing and waning hallucinations and mumbling speech throughout the day. A comprehensive gas chromatography/mass spectrometry urine analysis resulted 14 hours after arrival and was positive for diphenhydramine and doxylamine. Twenty hours from initial presentation, he required physical restraints during which IV access was lost and IM lorazepam 6 mg was administered.

The patient was demonstrating signs and symptoms consistent with antimuscarinic toxidrome, including agitation, combativeness, and delirium along with peripheral findings. Toxicology recommended reversal with physostigmine; however, the antidote was unavailable due to a medication shortage. Oral rivastigmine 0.75 mg was instead administered 24 hours post-initial presentation and 26 hours after his OTC dose at home. His agitation and delirium significantly improved over the next 6 hours post-rivastigmine administration, with only one IM lorazepam dose administered within 2 hours following rivastigmine, but otherwise precluding further benzodiazepine dosing. On hospital day (HD) 2, he was drowsy but able to answer simple questions and follow commands. No adverse effects occurred following rivastigmine administration. His delirium resolved within 24 hours post-rivastigmine administration with a return to baseline mentation by HD 3. The remainder of his clinical course involved treatment for mild rhabdomyolysis with IV fluid hydration. He was discharged on HD 4.

## DISCUSSION

Physostigmine is the preferred agent to reverse central antimuscarinic delirium; however, due to its shortage, other therapeutic options warrant investigation. Benzodiazepines, while often used to control agitation, do not address reversing the underlying muscarinic acetylcholine receptor blockade. Further, increasing doses and/or protracted use of parenteral benzodiazepines can lead to oversedation, hypotension, respiratory depression, and induced delirium [1, 3, 4].

While physostigmine and benzodiazepines are considered first-line agents for the pharmacological management of antimuscarinic delirium, overlapping drug shortages across the United States have impacted parenteral supplies of these medications. This has interfered with clinicians’ abilities to substitute one medication for another, including substitutions within the benzodiazepine drug class, and thus may impact treatment timeliness. Consequently, clinicians must consider use of second-line agents, including non-physostigmine cholinesterase inhibitors like rivastigmine. However, medication substitutions for treatment of antimuscarinic delirium may introduce the risk of adverse patient outcomes stemming from multifactorial contributors. These can include a lack robust indication-specific safety and efficacy data; lack of clinician familiarity with administration, dosing, pharmacokinetics and adverse effects; and unestablished dosing schedules. This can result in iatrogenic medication errors, undertreatment, adverse drug effects and ultimately negatively impact patient care in both emergency and critical care settings [5, 6].

Few reports describe rivastigmine use for antimuscarinic delirium. Van Kernebeck *et al*. used oral rivastigmine (1.5 mg) in an adult male exhibiting antimuscarinic delirium from procyclidine intoxication; he improved within 6 hours and returned to baseline within 24 hours with no relapse or adverse effects [2]. Hughes *et al*. treated an adult female with a more aggressive course of oral rivastigmine (12 mg) following acute diphenhydramine toxicity with symptom improvement within 2 hours, complete resolution within 24 hours, and no adverse effects or recurring antimuscarinic toxicity [7]. Transdermal rivastigmine use has been described in the treatment of psychotic symptoms from non-pharmaceutical scopolamine intoxication in six patients [8].

Rivastigmine may confer potential therapeutic benefits over physostigmine with the latter’s widespread shortage. These include its slower rate of penetration into the central nervous system, longer duration of action thereby reducing dosing frequency, theoretical potential for less severe side effects, and multiple drug delivery formulations [2, 7]. It may also be favorable when prolonged delirium is anticipated. Its transdermal formulation also provides a potentially useful delivery mechanism when agitation and delirium preclude oral drug administration. Limited pediatric data (ages 10–17 years) demonstrate that oral rivastigmine (1.5-4.5 mg/day) is well-tolerated in non-toxic patients [9]. Prospective randomized controlled trials comparing rivastigmine to current pharmacologic alternatives (i.e. benzodiazepines) are warranted to evaluate its safety, efficacy, and clinical utility.

Limitations include the lack of a clear temporal relationship between rivastigmine administration and improvement in the patient’s antimuscarinic delirium. The administration of benzodiazepines may have mitigated the degree of symptoms.

Rivastigmine is a potential alternative treatment for antimuscarinic delirium considering the national physostigmine shortage. Prospective trials are needed to appraise its utility and safety and to establish a temporal relationship with symptom reversal.
